# Neurofilament light predicts neurological outcome after subarachnoid haemorrhage

**DOI:** 10.1093/brain/awaa451

**Published:** 2021-01-31

**Authors:** Patrick Garland, Matt Morton, Ardalan Zolnourian, Andrew Durnford, Ben Gaastra, Jamie Toombs, Amanda J Heslegrave, John More, Henrik Zetterberg, Diederik O Bulters, Ian Galea

**Affiliations:** 1 Clinical Neurosciences, Clinical and Experimental Sciences, Faculty of Medicine, University of Southampton, Southampton, UK; 2 Wessex Neurological Centre, University Hospital Southampton NHS Foundation Trust, Southampton, UK; 3 UK Dementia Research Institute, University College London, UK; 4 Department of Neurodegenerative Disease, UCL Institute of Neurology, Queen Square, London, UK; 5 R&D, Bio Products Laboratory Limited, Elstree, Hertfordshire, UK; 6 Department of Psychiatry and Neurochemistry, Institute of Neuroscience and Physiology, The Sahlgrenska Academy at the University of Gothenburg, Mölndal, Sweden; 7 Clinical Neurochemistry Laboratory, Sahlgrenska University Hospital, Mölndal, Sweden

**Keywords:** neurofilament light, subarachnoid haemorrhage, haemoglobin, haptoglobin, glymphatic system

## Abstract

To improve outcome prediction following subarachnoid haemorrhage (SAH), we sought a biomarker integrating early brain injury and multiple secondary pathological processes in a prospective study of 42 non-traumatic SAH patients and 19 control individuals. Neurofilament light (NF-L) was elevated in CSF and serum following SAH. CSF and serum NF-L on Days 1–3 post-SAH strongly predicted modified Rankin score at 6 months, independent of World Federation of Neurosurgical Societies (WFNS) score. NF-L from Day 4 onwards also had a profound impact on outcome. To link NF-L to a SAH-specific pathological process, we investigated NF-L’s relationship with extracellular haemoglobin. Most CSF haemoglobin was not complexed with haptoglobin, yet was able to be bound by exogenous haptoglobin i.e. haemoglobin was scavengeable. CSF scavengeable haemoglobin was strongly predictive of subsequent CSF NF-L. Next, we investigated NF-L efflux from the brain after SAH. Serum and CSF NF-L correlated positively. The serum/CSF NF-L ratio was lower in SAH versus control subjects, in keeping with glymphatic efflux dysfunction after SAH. CSF/serum albumin ratio was increased following SAH versus controls. The serum/CSF NF-L ratio correlated negatively with the CSF/serum albumin ratio, indicating that transfer of the two proteins across the blood–brain interface is dissociated. In summary, NF-L is a strong predictive marker for SAH clinical outcome, adding value to the WFNS score, and is a promising surrogate end point in clinical trials.

## Teaser

Garland *et al.* show that neurofilament light levels predict long-term neurological outcomes following subarachnoid haemorrhage, if measured in CSF or serum within the first 72 hours. The measure improves accuracy of outcome prediction after the World Federation of Neurosurgical Societies score has been taken into account.

## Introduction

Subarachnoid haemorrhage (SAH) leads to an increase in intracranial pressure, transient cerebral ischaemia, and a subsequent prolonged multifactorial insult including neurotoxicity from haemoglobin released from the blood clot, delayed cerebral ischaemia, and inflammation (Macdonald, 2014). To predict long-term outcome in this patient population, it will be necessary to understand the contributions of early brain injury and subsequent pathological events, particularly from the prolonged exposure of the brain to haemoglobin and inflammatory molecules. Clinically, early brain injury can be assessed using the World Federation of Neurosurgical Societies (WFNS) score (Teasdale *et al.*, 1988), which can also partially predict long-term neurological outcome. In the largest study of outcome prediction to date, a number of predictors were tested including age, WFNS grade, premorbid history of hypertension, Fisher scale, aneurysm size, aneurysm location, and treatment modality (Jaja *et al.*, 2018). WFNS only explained a minor proportion of variance in outcome, and the contribution of the other predictors was substantially lower, by one or two orders of magnitude.

In trying to explain a larger proportion of outcome variance, we took advantage of neurofilament light (NF-L), an intracellular neuronal protein released during damage to neurons. NF-L can be detected in both CSF and serum and is a well-established biomarker for neuronal injury in neurodegenerative and neuroinflammatory conditions (Simren *et al.*, 2020). Since NF-L release can integrate neuronal damage from early brain injury as well as a number of delayed secondary pathological processes occurring subsequent to early brain injury, we hypothesized that NF-L can predict outcome after SAH, independent of WFNS.

## Materials and methods

### Sample collection

This study had ethical (National Research Ethics Committee approval numbers 11/SC/0204 and 12/SC/0666) and institutional (ERGO 41084 and ERGO 10492) approvals. Paired CSF and serum were collected daily on the first 3 days and on alternate days thereafter over a 14-day period post-ictus in 44 patients following original Fisher grade 3–4 (Fisher *et al.*, 1980) non-traumatic SAH with an external ventricular drain *in situ*, until this was removed. WFNS ranged between 1 and 5. Only 42 patients contributed to this study due to early removal of the external ventricular drain. Fresh CSF was collected after discarding a CSF volume equal to the dead space of the tubing. Control CSF was from 19 individuals undergoing lumbar puncture for non-inflammatory/haemorrhagic/degenerative neurological conditions, subsequently found to have normal CSF (glucose, protein, cell count and microbiological assessment). CSF was centrifuged at 1500 relative centrifugal force (RCF) for 10 min at 21°C and frozen within 1 h of sampling. Blood samples were routinely taken on the same days as the CSF samples. SAH patients were followed up at 6 months to determine clinical outcome as assessed by the modified Rankin Scale (mRS) (Quinn *et al.*, 2009) by researchers trained in using this scale.

### CSF neurofilament light assay

CSF NF-L was measured by ELISA (UmanDiagnostics). Briefly, samples were thawed at 21°C, and centrifuged at 1750 RCF for 5 min at 21°C. Samples were diluted 1:2 with sample diluent and added in duplicate to microplate wells coated with a monoclonal capture antibody specific for NF-L. Samples were then incubated with a biotinylated anti-NF-L monoclonal detection antibody. The detection complex was completed with the addition of horseradish peroxidase-labelled streptavidin and tetramethylbenzidine (TMB) substrate. Sample concentrations were calculated from a standard curve, fitted using a four-parameter logistic curve. The intra-assay coefficient of variation (CVs) was 1.8% and the inter-assay CV was 10.9%, calculated according to ISO 5725-2 standards.

### Serum neurofilament light assay

Serum NF-L was measured by single molecule array (Simoa) on an HD-1 analyser (Quanterix), according to the manufacturer’s instructions (Lot: 501832). Briefly, samples were thawed at 21°C, vortexed, and centrifuged at 10 000 RCF for 5 min at 21°C. Onboard the instrument, samples were diluted 1:4 with sample diluent and bound to paramagnetic beads coated with a capture antibody specific for human NF-L. NF-L bound beads were then incubated with a biotinylated anti-NF-L detection antibody in turn conjugated to streptavidin-β-galactosidase complex that acts as a fluorescent tag. Subsequent hydrolysis reaction with a resorufin β-d-galactopyranoside substrate produces a fluorescent signal proportional to the concentration of NF-L present. Duplicate measurements were taken of each sample. Sample concentrations were calculated from a standard curve, fitted using a four-parameter logistic curve. Intra-assay CVs were <11%, as determined by two quality controls (low and high concentration) according to ISO 5725-2 standards.

### CSF haemoglobin assay

Ultra-performance liquid chromatography (UPLC) was used to separate CSF components in a Tris-saline mobile phase, coupled to absorbance measurement at 415 nm to identify haem-containing species (Garland *et al.*, 2020). Haemoglobin was measured in CSF before and after saturating with haptoglobin (Bio Products Laboratory Limited) to measure haptoglobin-scavengeable and haptoglobin-non-scavengeable uncomplexed haemoglobin.

### CSF/serum albumin quotient

Albumin in matched CSF and serum samples was measured using a spectrometric assay run on a Beckman Coulter clinical chemistry analyser. The assay relies on the reaction of albumin with Bromocresol Purple to form a coloured complex; absorbance is measured at 600 nm to obtain an albumin measurement. Lumbar CSF albumin values were corrected by a factor of 2.2 (Weisner and Bernhardt, 1978) to enable comparison with ventricular CSF albumin values.

### Statistical analysis

Statistical analysis and graph preparation were performed using SPSS (v24) and GraphPad Prism (v7.01), with data expressed as median ± interquartile (IQR), mean ± standard error, or 95% confidence intervals (CI). The NF-L reference range was defined as the mean ± 1.96 standard deviations (SD) of the control population log-normal distribution. Area-under-the-curve (AUC) analysis was undertaken using the trapezoid method, and normalized per day. Normality and heteroscedasticity were routinely determined across all datasets. Where necessary, logarithmic transformation was used to normalize data. Model comparison for multivariate linear and ordinal regressions was performed using the F-test. In regression modelling, WFNS was dichotomized into low grade (i.e. 1–3) and high grade (i.e. 4–5). In ordinal regression, the proportional odds assumption was fulfilled. The Nagelkerke’s pseudo R^2^ value was used. Alpha (α), the probability of a type I error, was 0.05. Two-tailed hypotheses were considered throughout.

### Data availability

The data that support the findings of this study are available from the corresponding author, upon reasonable request, subject to institutional agreements and ethical approvals.

## Results

### Neurofilament light rises in both CSF and serum after subarachnoid haemorrhage

**Figure 1:**
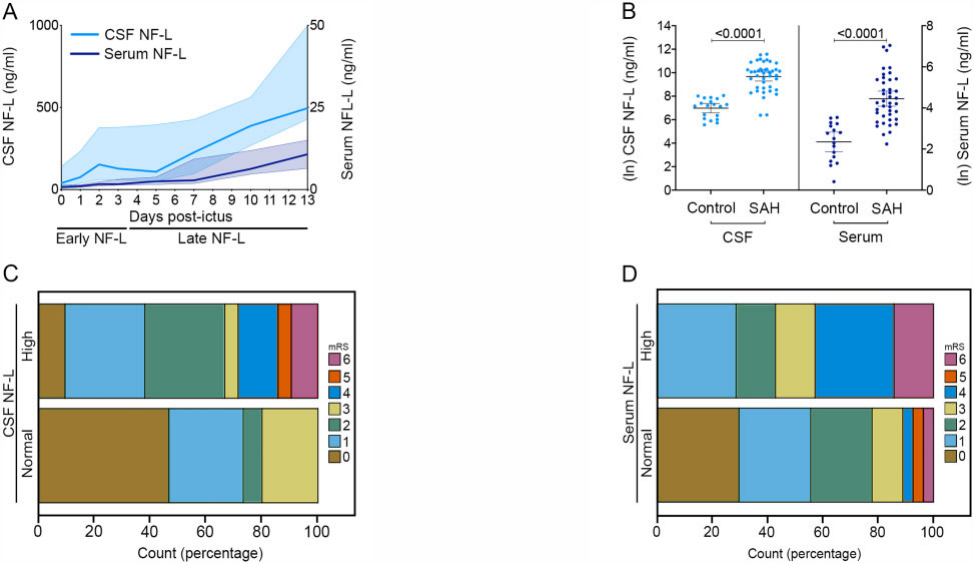
**CSF and serum NF-L predict long-term neurological outcome after SAH.** (**A**) Serial CSF and serum NF-L over a 2-week period following SAH [median ± IQR (shaded zone)]. (**B**) In both CSF and serum, the maximum NF-L observed for each patient over the 2-week sampling period was significantly greater than the control population (mean ± 95% CI; unpaired *t*-test, *P *<* *0.0001. CSF: control = 19, SAH = 42. Serum: control = 17, SAH = 41). (**C**) Ordinal logistical regression shows high CSF NF-L between Days 1–3 predicts poor outcome at 6 months on the mRS (odds ratio 4.3, 95% CI 1.1–16.2; *P* = 0.031, *n *=* *24 and 12 for high and normal CSF NF-L, respectively). (**D**) Ordinal logistic regression shows high serum NF-L between Days 1 and 3 predicts poor outcome at 6 months on the mRS (odds ratio 2.3, 95% CI 0.6–6.6; *P* = 0.04, *n *=* *7 and 27 for high and normal serum NF-L, respectively). High CSF and serum NF-L were defined as higher than the upper limit of the respective control population-based reference ranges. The NF-L reference range was defined as the mean ± 1.96 SD of the control population log-normal distribution.

Demographic and clinical characteristics are shown in Supplementary Table 1. CSF NF-L exhibited a small peak centred around Day 2 (Fig. 1A), following which both CSF and serum NF-L concentrations gradually increased over the 2-week sampling period (Fig. 1A). Compared to control NF-L levels, the maximum observed NF-L level in both CSF and serum was significantly greater (Fig. 1B). This is despite the fact that CSF NF-L is physiologically lower in ventricular CSF compared to lumbar CSF (Jeppsson *et al.*, 2013). Maximum CSF and serum NF-L were not significantly different between patients undergoing clipping, coiling or supportive management in an analysis of covariance (ANCOVA) controlling for age and WFNS (*P *=* *0.926 and *P *=* *0.511 for CSF and serum NF-L, respectively) (Supplementary Fig. 1).

### Early neurofilament light rise in both CSF and serum predicts neurological outcome after subarachnoid haemorrhage

In view of the NF-L peak at Day 2, we first investigated whether early NF-L measurement between Days 1 and 3 could explain additional variance in outcome alongside WFNS. Multivariable ordinal logistical regression was conducted, with mRS at 6 months post-ictus as the dependent variable, and NF-L as predictor, controlling for WFNS. Age, gender, premorbid history of hypertension, Fisher scale, size and location of ruptured aneurysm lumen, and treatment modality were not significant covariates in univariate analysis. High CSF and serum NF-L were defined as higher than the upper limit of the respective control population-based reference ranges. High CSF NF-L significantly predicted a worse mRS (Fig. 1C), with an odds ratio of 4.3 (95% CI: 1.1–16.2, *P *=* *0.031) for poor outcome. The odds ratio for WFNS was 10.4 (95% CI: 2.2–47.8, *P *=* *0.003). CSF NF-L and WFNS independently explained the 6-month mRS, such that CSF NF-L improved model fit compared to WFNS alone (pseudo R^2^ values: WFNS: 0.306, CSF NF-L: 0.179, WFNS + NF-L: 0.399, logit link function).

Similarly, high serum NF-L significantly predicted a poor neurological outcome at 6 months post-ictus (Fig. 1D), with an odds ratio of 2.3 (95% CI: 0.6–6.6, *P *=* *0.04). The odds ratio for WFNS was 3.2 (95% CI: 0.9–9.9, *P *=* *0.015). Serum NF-L and WFNS independently explained the 6-month mRS, such that serum NF-L improved model fit compared to WFNS alone (pseudo R^2^ values: WFNS: 0.225, serum NF-L: 0.128, WFNS + NF-L: 0.311, negative log-log link function).

Both CSF and serum NF-L independently predicted outcome when added to the Jaja core model predictors (Jaja *et al.*, 2018) (*P *=* *0.035 and *P *=* *0.037 for CSF and serum NF-L, respectively), improving model fit (pseudo R^2^ values: Jaja core model: 0.290, Jaja core model + CSF NF-L: 0.367, Jaja core model + serum NF-L: 0.348, negative log-log link function).

### Late neurofilament light rise in both CSF and serum predicts neurological outcome after subarachnoid haemorrhage

In view of the prolonged rise in NF-L from Day 4 onwards (Fig. 1A), we next studied whether the AUC (normalized per day) for NF-L from Day 4 onwards was related to outcome, independent of WFNS. To be able to compare the relative impact of early and late NF-L levels on outcome and to avoid bias resulting from patients who had an indwelling ventricular catheter for longer than others and therefore provided late samples, this analysis was performed on the maximum number of patients with samples in both early (Days 1–3) and late (Days 4 onwards) time epochs: 23 patients had data-points stretching between Days 2 and 7.

Multivariable ordinal regression controlling for WFNS showed that NF-L level in the late epoch contributed to outcome additional to WFNS, whether it was assessed in CSF (odds ratio: 4.6, 95% CI: 1.6–13.4, *P *=* *0.005) or serum (odds ratio: 2.4, 95% CI: 1.1–5.4, *P *=* *0.03). However, when both early and late CSF NF-L AUC were analysed together, only late CSF NF-L retained significance (odds ratio: 3.9, 95% CI: 1.3–11.8, *P *=* *0.017).

Late CSF and serum NF-L AUC were higher in patients with radiological evidence of infarction, though this did not reach significance in an ANCOVA controlling for age and WFNS (*P *=* *0.720 and *P *=* *0.184 for CSF and serum NF-L, respectively) (Supplementary Fig. 2).

### Scavengeable haemoglobin is correlated with the brain injury marker neurofilament light

To substantiate the relationship of NF-L to pathological processes that are (i) specific to SAH; (ii) delayed; and (iii) potentially reversible, we selected extracellular haemoglobin as an example for several reasons. First, haemoglobin is specific to SAH. Second, it is released gradually and its CSF concentration increases with time during the first 2 weeks post-SAH, as clot lysis proceeds (Durnford *et al.*, 2015). Third, haemoglobin neurotoxicity can be neutralized by intraventricular treatment with haptoglobin (Garland *et al.*, 2020), unless it undergoes oxidation (Faivre *et al.*, 1998), which prevents its binding to haptoglobin and renders it ‘unscavengeable’. Establishing a relationship between NF-L and scavengeable haemoglobin would indicate that NF-L could be used as a surrogate biochemical marker of outcome in clinical trials of intrathecal haptoglobin treatment.

**Figure 2:**
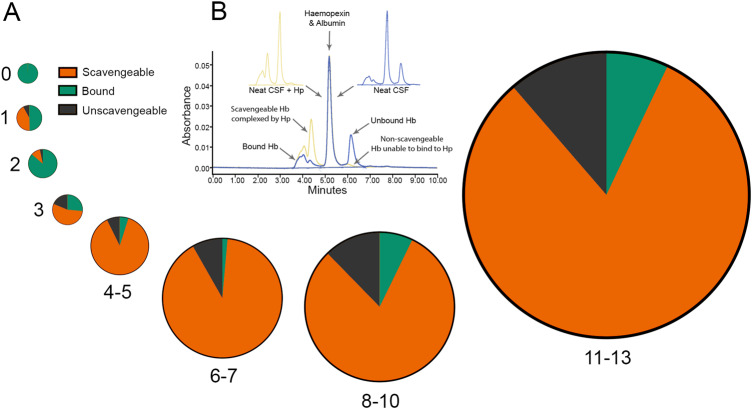
**Scavengeable haemoglobin is elevated in the CSF of SAH patients.** (**A**) Pie charts representing the relative proportions of bound, scavengeable and unscavengeable haemoglobin over the 2-week sampling period. Increasing diameter reflects increasing total haemoglobin. Days and day groupings indicated in the panel. (**B**) Size-exclusion chromatography to identify haemoglobin (Hb) species. Neat or haptoglobin (Hp)-saturated samples reveal haem-containing haemoglobin peaks in the 415 nm Soret band.

We quantified the fraction of scavengeable haemoglobin in the CSF after SAH (Fig. 2A). In the first 3 days most CSF haemoglobin was bound by haptoglobin derived from the plasma accompanying the SAH (Fig. 2B and Supplementary Fig. 3A). From Day 3 onwards, scavengeable haemoglobin predominated over non-scavengeable haemoglobin (Fig. 2B and Supplementary Fig. 3B and C); this increased to a maximum of 6.5 µM (median, IQR 1.179–15.390) between Days 11–13. From Day 4 onwards, 83% of the haemoglobin present in the CSF following SAH was scavengeable, 10% was unscavengeable, and a small amount of haemoglobin, 7%, was already bound to endogenous haptoglobin (Fig. 2B and Supplementary Fig. 3).

**Figure 3:**
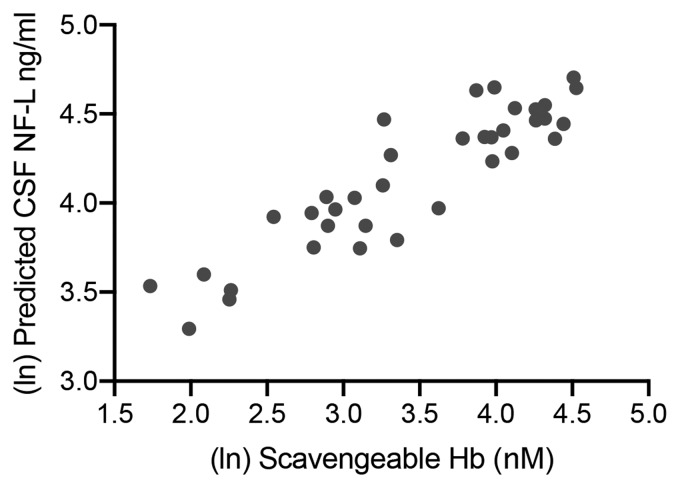
**Scavengeable haemoglobin is correlated with the brain injury marker NF-L.** Multiple linear regression was used to predict CSF NF-L levels using the highest preceding scavengeable haemoglobin (Hb), while controlling for WFNS, age and sex (R^2^ = 0.503, scavengeable haemoglobin *P *=* *0.0003; *n *=* *36); scavengeable haemaglobin plotted against model fitted (i.e. predicted) CSF NF-L values.

To investigate the relationship between CSF scavengeable haemoglobin and NF-L levels we used multiple linear regression, controlling for WFNS, age and sex (Fig. 3). With the maximum CSF NF-L value as the dependent variable, the highest preceding CSF scavengeable haemoglobin value was investigated as a predictor. CSF scavengeable haemoglobin level explained 50.3% of the variance in CSF NF-L level, with the *P*-value of the regression coefficient corresponding to the slope indicating a statistically significant dependence on scavengeable haemoglobin level (*P *=* *0.0003); neither age nor sex contributed significantly.

### Neurofilament light exchange across the blood–brain interface differs from that of albumin

**Figure 4:**
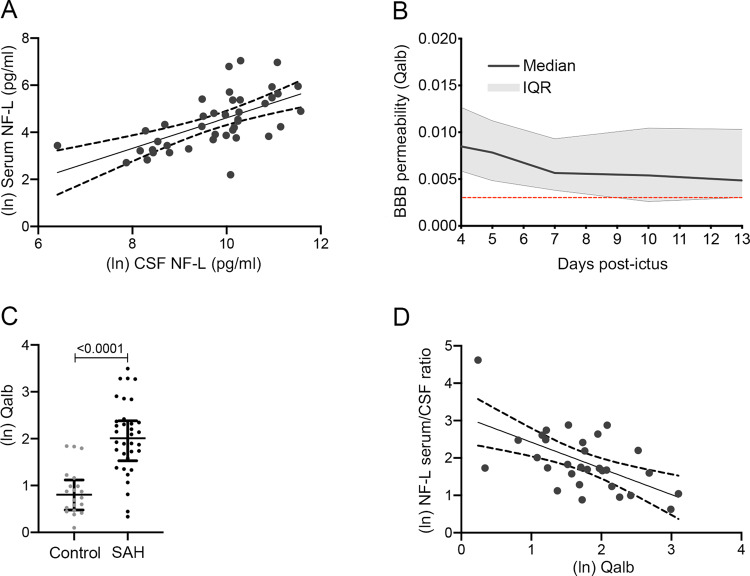
**Brain-to-blood NF-L efflux.** (**A**) Scatter plot of CSF NF-L versus serum NF-L reveals a significant Pearson r correlation (r = 0.62, *P *<* *0.0001, *n *=* *41). (**B**) Qalb is elevated from Day 4 until the end of the 2-week sampling period. The red line is the mean value for the control population. (**C**) The maximum Qalb for each patient over the 2-week sampling period is significantly greater than the control population (mean ± SEM; unpaired *t*-test, *P *<* *0.0001, *n *=* *20 and 34 for controls and SAH, respectively). (**D**) Scatter plot of Qalb versus the serum/CSF NF-L ratio reveals a significant negative Pearson *r* correlation (*r* = −0.58, *P *=* *0.0008, *n *=* *42).

A circulating biomarker, such as serum NF-L, would be of immense value following SAH, since CSF would only be accessible in a minority of patients. Serum NF-L concentration would be expected to follow CSF NF-L due to brain–blood efflux, and indeed this was the case with the maximum CSF NF-L predicting the subsequent maximum serum NF-L over the sampling period (Fig. 4A). The serum/CSF NF-L ratio represents the transfer of NF-L from CSF to blood. The serum/CSF NF-L ratio was lower in SAH versus control subjects (medians of 0.0057 versus 0.01, respectively, Mann-Whitney *P *=* *0.024), suggesting glymphatic efflux dysfunction after SAH.

NF-L (62 kDa) has a similar molecular weight to albumin (60 kDa), so we investigated whether NF-L efflux and albumin influx across the blood–brain interface, albeit in different directions, were synchronized, since this may provide insight into the routes of exchange of these two molecules. Albumin influx from the blood into the CSF occurs via the choroid plexus and the blood–brain barrier (Thompson, 2005). Since albumin is not synthesized in the CNS, the ratio between CSF and serum albumin concentration—the albumin quotient, or Qalb, is used as a proxy for blood–brain barrier permeability (Tibbling *et al.*, 1977). An increase in this ratio indicates that there is increased blood–brain barrier permeability to molecules of similar size to albumin.

There is evidence that blood–brain barrier permeability increases after SAH (Galea *et al.*, 2012). To investigate changes in blood–brain barrier permeability, paired CSF and serum samples from SAH patients taken at Day 4 or later were assayed for albumin. Time points earlier than Day 4 were excluded since analysis at these time points would have been confounded by albumin that had entered the CSF with the bleed (Supplementary Fig. 4). Figure 4B shows there is a sustained elevation of Qalb after SAH from Day 4 onwards relative to the control population (red line). An unpaired *t*-test between the control population and the maximum Qalb in the SAH patients shows a highly significant difference (Fig. 4C).

NF-L may exit the brain into the circulation via a number of routes, including CSF drainage via the cribriform plate, nerve roots and dural lymphatics, the intramural peri-arterial pathway and reflux across the gliovascular membrane complex at the blood–brain barrier (Rasmussen *et al.*, 2018). If the blood–brain barrier, namely the gliovascular membrane complex at capillary level, was the route for NF-L efflux, one would expect a positive correlation between the serum/CSF NF-L ratio and Qalb. Hence we explored the relationship between the mean serum/CSF NF-L ratio and Qalb. This showed a significant negative correlation (Fig. 4D). This finding was robust to sensitivity analyses, which included using the maximum Qalb and the serum/CSF NF-L ratio on the same day, and adding CSF drainage volume as a covariate. In summary, NF-L exchange across the blood–brain interface differs from that of albumin. This phenomenon is not specific for SAH, since we reproduced the same observation in an independent set of samples from clinical neurochemistry practice, representing a wide range of serum/CSF NF-L ratios and Qalb results (Supplementary Fig. 5).

## Discussion

SAH results in a long-term deficit in neurological function, yet outcome prediction is challenging. Here we take the novel approach of combining WFNS as a clinical marker of early brain injury, and NF-L as an independent biomarker integrating multiple post-injury processes, to best model long-term neurological outcome in SAH patients. An early CSF or serum NF-L estimation, within the first 1–3 days after SAH, adds value to the WFNS, in the prediction of 6-month outcome following SAH. A serum biomarker is significantly advantageous over a CSF biomarker, since CSF may not be readily available in all patients. External ventricular drain placement is required for clinical management in only a third of patients (Galea *et al.*, 2017), and lumbar CSF drainage is not a mainstay of treatment.

This study extends and clarifies research examining neurofilament subunits, namely heavy (Petzold *et al.*, 2005, 2006; Lewis *et al.*, 2008) and light (Nylen *et al.*, 2006; Zanier *et al.*, 2011; Halawa *et al.*, 2018; Hviid *et al.*, 2020) chains, following SAH. These studies are summarized in Supplementary Table 2. NF-L is a cytoskeletal protein widely expressed in neurons, which is emerging as a robust marker of brain injury (Gaetani *et al.*, 2019). Presymptomatic disease, such as mild cognitive impairment (Kern *et al.*, 2019), may be theoretically possible in some control individuals. Hence in the idealistic scenario where all control individuals are guaranteed to be free of subclinical neurodegenerative disease, the difference between SAH and controls would be even more accentuated.

The early prognostic effect of NF-L suggests that its release is likely to be linked to early brain injury. The latter triggers a number of prolonged processes such as inflammation and oxidative stress while other processes have a delayed onset such as delayed cerebral ischaemia, haemoglobin toxicity, ferrotoxicity and other as yet unidentified mechanisms. Hence it is not surprising that CSF NF-L AUC from Day 4 onwards had a bigger impact on the 6-month outcome, compared to CSF NF-L AUC on Days 1–3. While it is not clinically practical to measure NF-L levels repeatedly, AUC analysis here provided biological insight. In particular, the dramatic contribution of CSF NF-L AUC in the late epoch suggested that significant neuronal damage occurs in a delayed manner, and this impacts significantly on outcome. It is very possible that early brain injury directly following SAH may interact with secondary processes to determine outcome.

One of the prolonged secondary pathological processes likely to be integrated in the NF-L rise after SAH, is the gradual release of haemoglobin from the clot. Extracellular haemoglobin is directly neurotoxic (Garland *et al.*, 2020) and also triggers tertiary processes such as vasoconstriction, inflammation and iron deposition (Bulters *et al.*, 2018), which themselves propagate neuronal damage. Intracranial haptoglobin treatment has potential to prevent these effects (Garland *et al.*, 2020). To determine whether NF-L could be used as a surrogate marker for treatment trials of haptoglobin, we considered the proportion of haemoglobin able to bind haptoglobin. This ‘scavengeable’ haemoglobin makes up most of the CSF haemoglobin after Day 3 post-SAH. Here we show that peak CSF NF-L was strongly associated with preceding peak CSF ‘scavengeable’ haemoglobin, suggesting that CSF NF-L could be useful as a surrogate marker for efficacy of intracranial haptoglobin in phase II trials. It also demonstrates a link between NF-L and a delayed pathological process such as haemoglobin toxicity.

Not surprisingly, the clinical significance of raised serum NF-L followed that of CSF NF-L. A number of factors are likely to affect the relationship between CSF and serum NF-L, as NF-L generated intrathecally is transferred to the circulation, including blood–brain transfer rate and circulating half-life. Nevertheless, serum NF-L correlated with CSF NF-L, in keeping with the prognostic value of NF-L in both compartments.

The blood–brain barrier was compromised after SAH, as confirmed by the high Qalb in SAH patients compared to control subjects; this has been previously reported (Galea *et al.*, 2012). After experimental SAH, damage to the capillary basement membrane (Scholler *et al.*, 2007), increased pinocytosis and opening of tight junctions (Doczi, 1985) causes blood–brain barrier breakdown.

The serum/CSF NF-L ratio can be considered to be a marker of the rate of solute drainage from the brain. This ratio was decreased in SAH patients versus controls, in keeping with glymphatic efflux dysfunction, which has been recently demonstrated in an animal model of SAH (Pu *et al.*, 2019). Surprisingly, brain–blood NF-L transfer correlated negatively with blood–brain albumin transfer. This novel observation was not specific to SAH, since we observed a similar pattern in an unselected population of neurological patients. Impaired glymphatic clearance in those with a damaged blood–brain barrier is a likely explanation—the co-occurrence of glymphatic dysfunction and blood–brain barrier disruption in brain pathology has been previously discussed and postulated (Verheggen *et al.*, 2018), and our observation is in keeping with this idea. It appears that NF-L and albumin use different routes of transfer across the blood–brain interface. Hence NF-L transfer from brain to blood does not occur across a leaky blood–brain barrier in the capillary bed, but rather other pathways such as CSF efflux across the cribriform plate, nerve roots sleeves and dural lymphatics, and interstitial fluid efflux pathways across paravascular routes (Rasmussen *et al.*, 2018).

The highlight of this work is the prognostic usefulness of CSF and serum NF-L in predicting clinical outcome after SAH. Further work is required to progress these findings in larger more generalizable cohorts, including SAH patients with lower Fisher grades. We introduce the novel concept of including a biomarker integrating an array of early and secondary processes, in predictive modelling of SAH outcome. NF-L is used here as an example; other radiological or biochemical markers may be better, or their addition may improve prediction. The use of such variables has potential to transform modelling of long-term clinical outcome following SAH.

## Supplementary Material

awaa451_Supplementary_Data

## Abbreviations

mRS = modified Rankin Scale; NF-L: neurofilament light
SAH: subarachnoid haemorrhage
WFNS: World Federation of Neurosurgical Societies
