# Supplementary material for: Neurofilament light predicts neurological outcome after subarachnoid haemorrhage
Source: Brain. 2021 Jan 31;144(3):761–8. doi: 10.1093/brain/awaa451 (PMC8041040; doi:10.1093/brain/awaa451)
Supplement: awaa451_Supplementary_Data [file awaa451_supplementary_data.pdf]

SUPPLEMENTARY MATERAL FOR:

Neurofilament light predicts neurological outcome after subarachnoid haemorrhage

Patrick Garland[1,*], Matt Morton[1,*], Ardalan Zolnourian[2], Andrew Durnford[2], Ben Gaastra[2], Jamie Toombs[3,4], Amanda J Heslegrave[3,4], John More[5], Henrik Zetterberg[3,4,6,7#], Diederik O Bulters[2,#], Ian Galea[1,2, ψ, #]

* shared primary authorship

[#] shared senior authorship

[ψ] corresponding author: I.Galea@soton.ac.uk

[1] Clinical Neurosciences, Clinical & Experimental Sciences, Faculty of Medicine, University of Southampton, Southampton, United Kingdom

[2] Wessex Neurological Centre, University Hospital Southampton NHS Foundation Trust, Southampton, United Kingdom

[3] UK Dementia Research Institute, University College London, UK

[4] Department of Neurodegenerative disease, UCL Institute of Neurology, Queen Square, London, UK

[5] R&D, Bio Products Laboratory Limited, Elstree, Hertfordshire, United Kingdom

[6] Department of Psychiatry and Neurochemistry, Institute of Neuroscience and Physiology, The Sahlgrenska Academy at the University of Gothenburg, Mölndal, Sweden

[7] Clinical Neurochemistry Laboratory, Sahlgrenska University Hospital, Mölndal, Sweden

Supplementary Figure 1. CSF and serum NF-L were not significantly different between patients who had surgery, coiling, or supportive management. Plot shows estimated marginal means (with 95% confidence intervals) for maximum CSF and serum NF-L from analyses of covariance controlling for age and WFNS ($F(2,37)=0.077$, $p=0.926$ for CSF NF-L and $F(2,36)=0.685$, $p=0.511$ for serum NF-L).

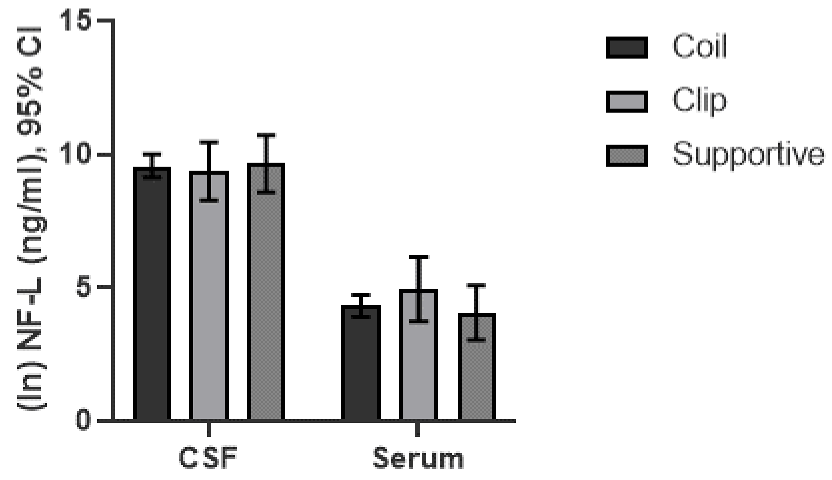

Supplementary Figure 2. Late CSF and serum NF-L area-under-the-curve estimated marginal means, adjusted for age and WFNS, in patients with radiological evidence of infarction. Plot shows estimated marginal means (with 95% confidence intervals) for late CSF and serum NF-L area-under-the-curve (normalized per day) from an analysis of covariance controlling for age and WFNS (F(1,28)= 0.131, p=0.720 for CSF NF-L and F(1,30)= 1.849, p=0.184 for serum NF-L).

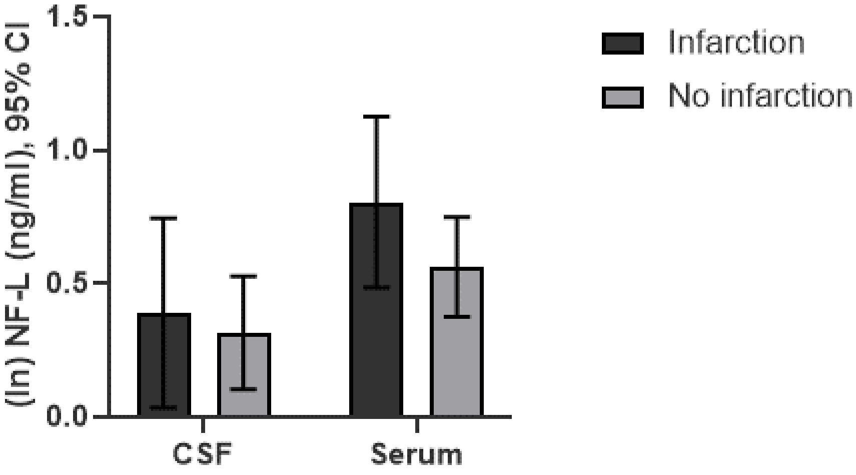

Supplementary Figure 3. UPLC characterisation of haemoglobin species accumulation following SAH. (**A**) Haemoglobin that is bound to endogenous Hp, 'Bound haemoglobin', plateaus after 3 days post-ictus. (**B**) Haemoglobin that can bind haptoglobin, 'scavengeable haemoglobin', gradually increases over the two-week sampling period. (**C**) Haemoglobin that cannot bind Hp, 'unscavengeable haemoglobin', is at minor amounts over the two-week sampling period. All data median ± interquartile range.

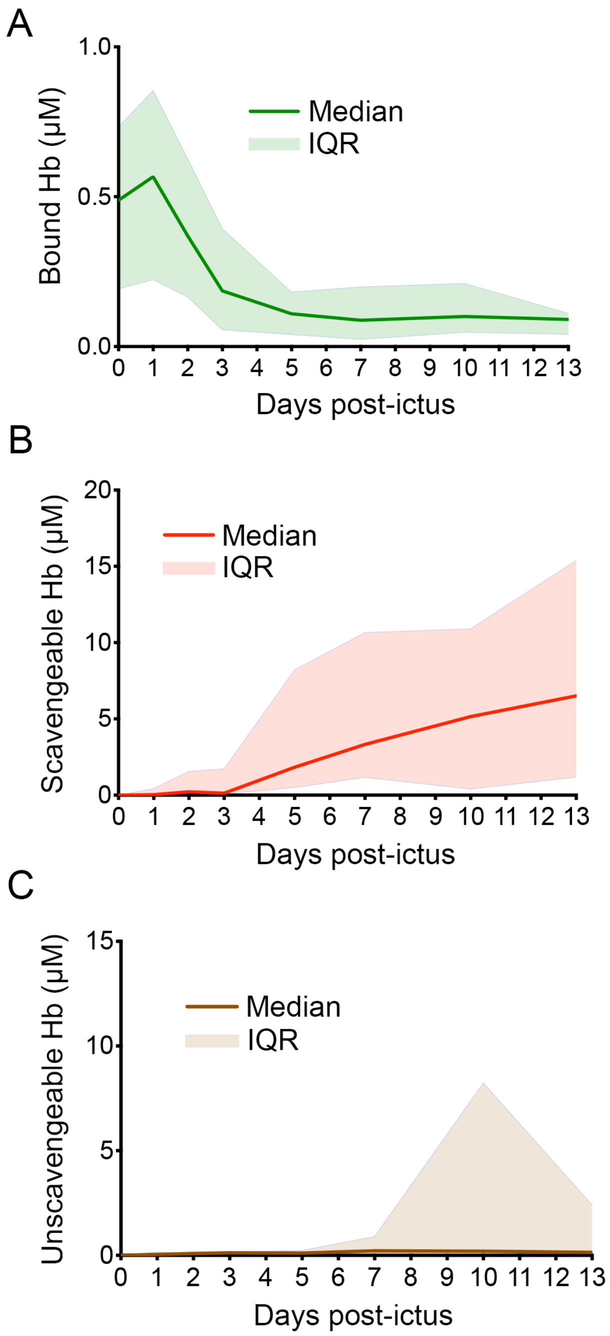

Supplementary Figure 4. Plasma proteins haptoglobin and albumin are present at a high concentration in the CSF in the first three days after SAH.

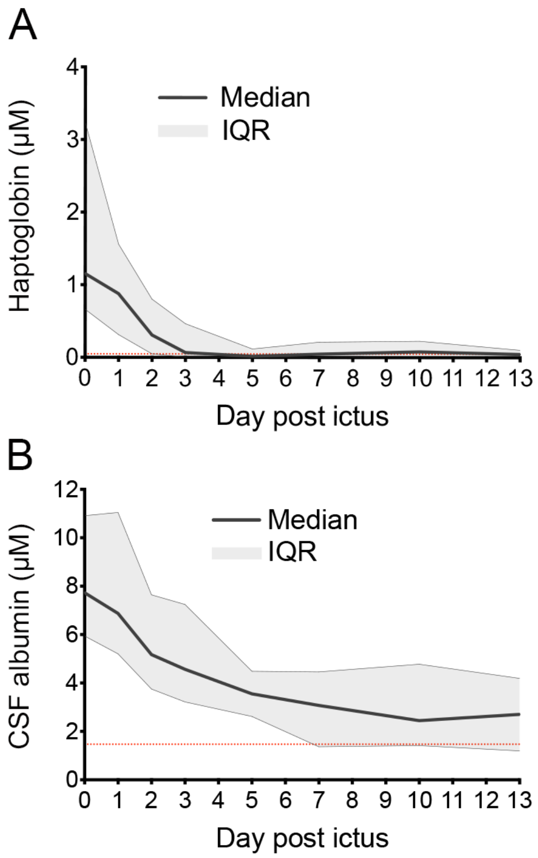

Supplementary Figure 5. Serum/CSF NF-L ratio correlates negatively with Qalb. Performed in an independent set of unselected samples from a clinical neurochemistry laboratory, representing a wide range of serum/CSF NF-L ratios and Qalb results (p=0.0035, r=-0.44).

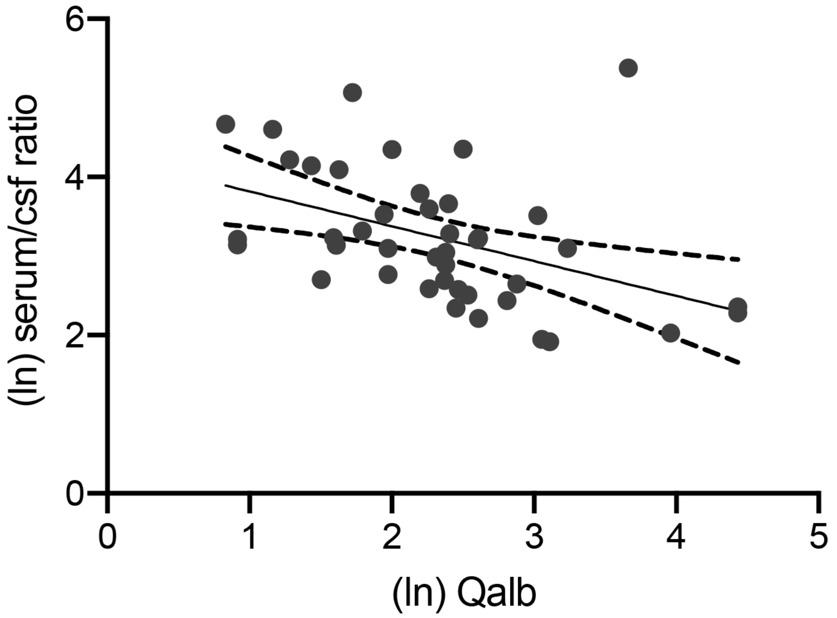

Supplementary Table 1. Clinical characteristics of included patients and controls

	Non-traumatic SAH patients	Control patients	p
Number	42	19	
Age (years)[a]	63 (21-82)	67 (21-92)	0.80[g]
Gender[b]			
Male	15 (35.7%)	5 (26.3%)	0.56[h]
Female	27 (64.3%)	14 (73.7%)	
Premorbid hypertension[b]			
Yes	18 (42.9%)		
No	24 (57.1%)		
Initial WFNS[b]			
1	4 (9.5%)		
2	8 (19%)		
3	3 (7.1%)		
4	20 (47.6%)		
5	7 (16.7%)		
Fisher grade[b]			
3	2 (4.8%)		
4	40 (95.2%)		
Delayed ischaemic neurological deficit[c]			
Yes	5 (12.2%)		
No	36 (87.8%)		
Radiological evidence of ischaemia[c]			
Yes	10 (24.4%)		
No	31 (75.6%)		
Aneurysmal management[b]			
Coiled	32 (76.2%)		
Clipped	5 (11.9%)		
Supportive	5 (11.9%)		
Aneurysm location[b,d]			
Anterior cerebral artery[e]	16 (38.1%)		
Middle cerebral artery	5 (11.9%)		
Posterior circulation[f]	14 (33.3%)		
Internal carotid artery	3 (7.1%)		
No vascular abnormality	4 (9.5%)		

a, median and range
b, number and %
c, information available for 41 patients
d, the ruptured aneurysm is indicated in cases with multiple aneurysms
e, included anterior cerebral, anterior communicating and pericallosal arteries
f, included posterior cerebral, posterior communicating, posterior inferior cerebellar and basilar arteries
g, Mann-Whitney test
h, Fisher's exact test

Supplementary Table 2. Summary of main characteristics of previous studies examining neurofilament subunits after SAH

			Garland et al	Petzold et al 2005	Petzold et al 2006	Nylen et al 2006	Lewis et al 2008	Zanier et al 2010	Halawa et al 2017	Vinter Bodker Hviid et al 2020
**PMID**			current study	15785235	16705199	16806706	18319731	20571038	29164612	31808039
**SAH patients (n)**	≥40	<40	42	10	17	48	30	35	19	44
**Controls within study (n)**	≥10	None	19	20	None	None	None	13	None	44
**Controls: neurodegeneration / inflammation excluded?**	Yes	No or N/A	Yes	No	N/A	N/A	N/A	Yes	N/A	Yes
**Biomarker**			NF-L	NF-H	NF-H	NF-L	NF-H	NF-L	NF-L	NF-L
**Source of CSF in SAH patients**	Done	Not done	EVD	EVD	EVD	LP	EVD	EVD	EVD	Not done
**Source of CSF in controls**	Done	Not done	Lumbar	Lumbar	Not done	Not done	Not done	Lumbar	Not done	Not done
**Paired CSF/serum in patients**	Yes	No	Yes	No	No	No	Yes	No	No	No
**Paired CSF/serum in controls**	Yes	No	Yes	No	No	No	No	No	No	No
**Sampling period (days post-ictus)**	Included days 1-3	After day 3 / Not defined	days 1-14	days 1-8	days 1-14	day 11 only	days 1-12	Not defined	days 4 and 10	4 hours and day 1
**CSF assay**	Done	Not done	ELISA	ELISA	ELISA	ELISA	ELISA	ELISA	ELISA	Not done
**Serum assay**	Done	Not done	SIMOA	Not done	Not done	Not done	ELISA	Not done	Not done	SIMOA
**Primary clinical outcome measure**	Studied	Not studied	6 months mRS	Not studied	3 month GOS	12 month GOSE	6 months GOSE	6 month GOS	DCI	day 30 mRS
**Association of primary outcome with CSF NF**	+	Not studied / detected	+	Not studied	+	+	Not studied	Not detected	Not detected	Not studied
**Association of primary outcome with serum NF**	+	Not studied /detected	+	Not studied	Not studied	Not studied	+	Not studied	Not studied	+
**Multivariable regression with WFNS**	Done	Not studied	Done	Not studied	Not studied	Not studied	Not studied	Not studied	Not studied	Done
**Outcome prediction: early versus late comparison**	Done	Not studied	Done	Not studied	Not studied	Not studied	Not studied	Not studied	Not studied	Not studied
**Mechanistic studies**	Done	Not studied	Done	Done	Not studied	Not studied	Done	Not studied	Not studied	Not studied
